# Interferon Beta-1a versus Combined Interferon Beta-1a and Oligodendrocyte-Specific FGFR1 Deletion in Experimental Autoimmune Encephalomyelitis

**DOI:** 10.3390/ijms232012183

**Published:** 2022-10-12

**Authors:** Ranjithkumar Rajendran, Vinothkumar Rajendran, Liza Gupta, Kian Shirvanchi, Darja Schunin, Srikanth Karnati, Mario Giraldo-Velásquez, Martin Berghoff

**Affiliations:** 1 Experimental Neurology, Department of Neurology, University of Giessen, Klinikstrasse 33, 35385 Giessen, Germany; 2 Institute of Anatomy and Cell Biology, University of Würzburg, Koellikerstrasse 6, 97080 Würzburg, Germany; 3 Department of Neurology, Sozialstiftung Bamberg, Buger Strasse 80, 96049 Bamberg, Germany

**Keywords:** FGFR1, interferon beta-1a, oligodendrocytes, EAE, multiple sclerosis

## Abstract

Recombinant beta interferons-1 (IFNβ-1) are used as first line therapies in patients with relapsing multiple sclerosis (MS), a chronic inflammatory and neurodegenerative disease of the CNS. IFNβ-1a/b has moderate effects on the prevention of relapses and slowing of disease progression. Fibroblast growth factors (FGFs) and FGF receptors (FGFRs) are known to play a key role in the pathology of MS and its model EAE. To investigate the effects of short-term treatment with s.c. IFNβ-1a versus the combined application of s.c. IFNβ-1a and oligodendrocyte-specific deletion of FGFR1 (*Fgfr1^ind^*^−/−^ mice) in MOG_35-55_-induced EAE. IFNβ-1a (30 mg/kg) was applied s.c. from days 0–7 p.i. of EAE in controls and *Fgfr1^ind^*^−/−^ mice. FGFR signaling proteins associated with inflammation/degeneration in MS/EAE were analyzed by western blot in the spinal cord. Further, FGFR1 in Oli-neu oligodendrocytes were inhibited by PD166866 and treated with IFNβ-1a (400 ng/mL). Application of IFNβ-1a over 8 days resulted in less symptoms only at the peak of disease (days 9–11) compared to controls. Application of IFNβ-1a in *Fgfr1^ind^*^−/−^ mice resulted in less symptoms primarily in the chronic phase of EAE. *Fgfr1^ind^*^−/−^ mice treated with IFNβ-1a showed increased expression of pERK and BDNF. In Oli-neu oligodendrocytes, treatment with PD166866 and IFNβ-1a also showed an increased expression of pERK and BDNF/TrkB. These data suggest that the beneficial effects in the chronic phase of EAE and on signaling molecules associated with ERK and BDNF expression are caused by the modulation of FGFR1 and not by interferon beta-1a. FGFR may be a potential target for therapy in MS.

## 1. Introduction

Multiple sclerosis (MS) is a chronic inflammatory and neurodegenerative disease of the central nervous system. Immigration of immune cells into the CNS causes damage of myelin sheaths and oligodendrocytes, and the failure of remyelination plays a key role in this disease [1,2]. Currently approved disease-modifying treatments (DMT) primarily have anti-inflammatory effects in the periphery but do not pass the blood–brain barrier [3]. This also applies to recombinant beta interferons-1 (IFNβ-1a/b), which were the first DMTs available for relapsing MS (RMS) and are still widely applied [3]. Used as a first-line therapy, IFNβ-1a/b has moderate effects on the frequency of relapses and disease progression [3,4,5]. IFNβ-1 acts through a broad spectrum of immunomodulatory effects such as the alteration of antigen presentation, induction of regulatory T cells, reduction of matrix metalloproteinases, and inhibition of autoreactive lymphocytes and Th17 cell differentiation [3,6,7]. Overall, new insights into the underlying mechanisms and cross talk of IFN-β in other signaling pathways may reveal new treatment strategies to exploit the beneficial effects of IFN-β in patients with MS.

Recent research suggests that fibroblast growth factor (FGF)/FGF-receptor (FGFR) signaling pathways modulate the pathogenesis of MS through inflammatory and myelinating effects [8,9,10,11,12]. In post-mortem brain tissue, FGF2+ oligodendrocytes are present in demyelinating lesions, and FGF2 is considered to be an inhibitor of the differentiation of oligodendrocyte progenitor cells (OPC), thus comprising (re-) myelination [10,13], but also exerts neuroprotective effects such as improved neurogenesis [10,13,14]. FGF9 inhibits myelination, induces expression of pro-inflammatory chemokines, and may have an impact on the differentiation of oligodendrocytes [10]. In contrast, FGF1 is assumed to promote remyelination [11]. Oligodendrocyte-specific deletion of FGFR1 in MOG_35-55_-induces experimental autoimmune encephalomyelitis (EAE), an established mouse model for MS, ameliorates the disease’s course, reduces inflammation in demyelinating lesions, and results in less myelin degeneration and higher axonal density [15,16]. These effects were accompanied by the increased phosphorylation of ERK1/2 and Akt, which is reported to increase myelination [17]. Furthermore, the enhanced expression of BDNF and its receptor TrkB, and a decrease in LINGO-1, which inhibits the differentiation of oligodendrocytes [18], was found. The relevance of FGF/FGFR signaling in EAE was further supported by similar findings after the deletion of oligodendrocyte-specific FGFR2 [9]. Collectively, these data indicate a significant regulation of FGF/FGFR signaling pathways in autoimmune diseases such as MS and EAE.

Recent studies suggest that there exists some crosstalk between FGF and IFNβ pathways. Mice lacking FGFR1 and FGFR2 in keratinocytes overexpress type-I IFN-stimulated genes (ISGs) [19]. In addition, FGF signaling suppresses the basal expression of interferon-stimulated genes (ISGs) in keratinocytes and intestinal epithelial cells [19]. In vitro experiments with keratinocytes show that FGF7 or FGF10 suppress IFN-induced ISG expression via FGFR mediated ERK1/2 and AKT signaling [19]. Further, FGF7 prevents the trafficking of IFN-stimulated STAT1 in human airway epithelia, thus preventing the transcription of several IFN-induced genes [20]. In contrast, in human fetal astrocytes infected with the Zika virus, the blocking of FGF2 signaling leads to the increased expression of IFNβ gene and ISGs [21]. In mouse fibroblast and human cancer cells, Ras/MEK/ERK activation suppresses the transcription of IFN-inducible genes by downregulating IRF1, a transcriptional activator of the IFNβ gene [22], whereas in mouse embryo fibroblasts infected with the myxoma virus, ERK1/2 signaling promotes type-I IFN expression by the activation of IRF3 [23]. In human renal carcinoma cells, type-I IFN downregulates the expression of FGF2 [24]. Taken together, FGF/FGFR signaling by ERK/Akt is involved in Type-I IFN responses, though the exact mechanism of the crosslink remains elusive. 

The present study aimed to investigate the effects of short-term treatment with s.c. IFNβ-1a (30 mg/kg over 8 days) versus the combined application of s.c. IFNβ-1a and the oligodendrocyte-specific deletion of FGFR1 (*Fgfr1^ind^*^−/−^ mice) in MOG_35-55_-induced EAE. Compared with the treatment of IFNβ-1a in controls, the application of IFNβ-1a in *Fgfr1^ind^*^−/−^ mice significantly reduced symptoms in the chronic phase of EAE, which is characterized by neurodegeneration. Effects on the disease’s course were accompanied by increased ERK phosphorylation and the increased expression of BDNF in the spinal cord, associated with neuroregeneration. In vitro, the combined treatment of Oli-neu oligodendrocytes with the FGFR1-inhibitor PD166866 and IFNβ-1a also resulted in the increased phosphorylation of ERK and increased BDNF/TrkB expression. These data suggest that the effects seen in chronic EAE are mediated by the inhibition of FGFR and not treatment with the beta interferon.

## 2. Results

### 2.1. Beneficial Effects in the Chronic Phase of EAE Are Due to the Conditional Deletion of FGFR1 

**Figure 1:**
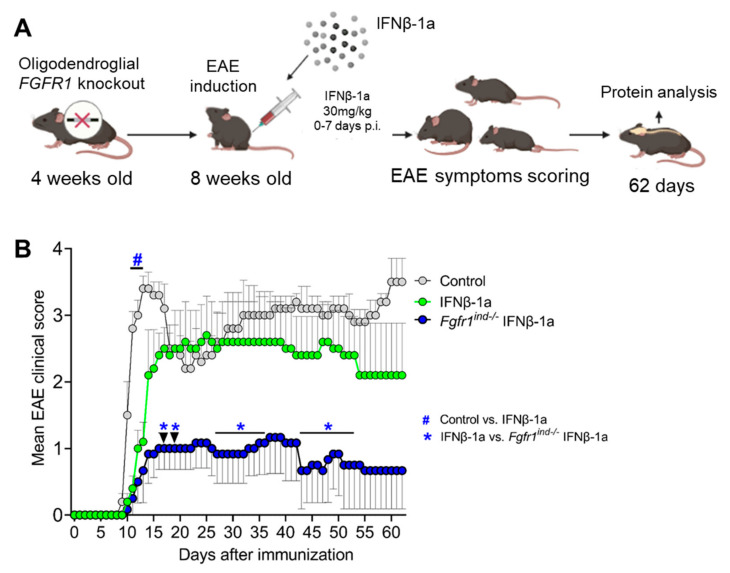
Experimental design and the clinical outcome of EAE scores. (**A**) Tamoxifen-induced FGFR1 knockout was created in 4-week-old mice, and at 8 weeks of age, control and *Fgfr1^ind^*^−/−^ mice were immunized subcutaneously with MOG_35-55_-peptide emulsified in CFA. Both mice group received 30 mg/kg IFNβ-1a from day 0–7 post-EAE-induction, and the development of neurological symptoms was monitored for 62 days. (**B**) A mean EAE clinical score of untreated control mice (*n* = 5), IFNβ-1a-treated control mice (*n* = 5) compared with IFNβ-1a-treated *Fgfr1^ind^*^−/−^ mice (*n* = 6). IFNβ-1a-treated *Fgfr1^ind^*^−/−^ mice showed a significant reduction of clinical symptoms. Data are expressed as the mean + SEM for each group. * *p* < 0.05.

To compare the effects of IFNβ-1a in controls and in *Fgfr1^ind^*^−/−^ mice on the clinical course of MOG_35-55_-induced EAE, both groups received IFNβ-1a (30 mg/kg) from day 0 until day 7 p.i. (Figure 1B). Compared with the untreated controls, s.c. application of IFNβ-1a resulted in less symptoms at the peak of disease (days 11–13 p.i.; *p* < 0.05). *Fgfr1^ind^*^−/−^ mice, which had received IFNβ-1a, showed a less severe disease course after the peak of disease (days 17, 19 p.i.; *p* < 0.05) and its chronic phase (day 27–36, 43–53 p.i.; *p* < 0.05) compared to IFNβ-1a treated controls (Figure 1B). Although not statistically significant, controls on IFNβ-1a had a mean EAE score of 2.1 ± 0.46, and *Fgfr1^ind^*^−/−^ mice on IFNβ-1a had a mean score of 0.66 ± 0.42 at the end of the experiment (*p* = 0.056). These data suggest that IFNβ-1a applied to prevent relapses only shows short-term effects at the peak of disease in MOG_35-55_-induced EAE. The effects seen after the peak of disease and in the chronic phase of EAE are due to conditional FGFR1 gene deletion. 

### 2.2. Increased Expression of pERK and BDNF in Fgfr1^ind−/−^ Mice 

**Figure 2:**
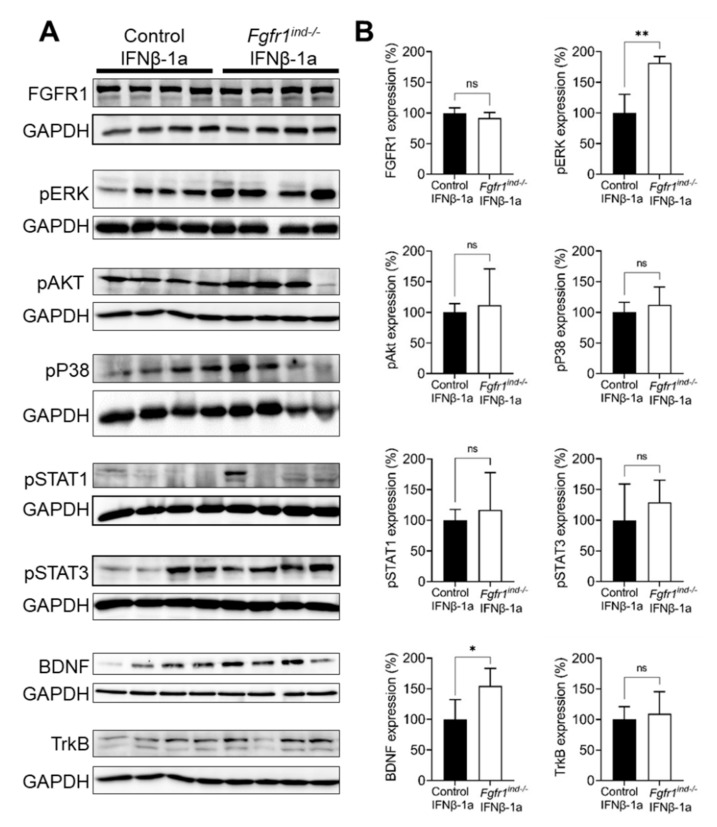
Protein expression of FGFR signaling and TrkB/BDNF in spinal cord lysates at day 62 p.i. (**A**,**B**). Protein expression of FGFR1, pAKT, pP38, pSTAT1/3, and TrkB were not different between IFNβ-1a-treated controls and IFNβ-1a-treated *Fgfr1^ind^*^−/−^ mice. The increased phosphorylation of ERK and increased BDNF protein expression was found in IFNβ-1a-treated *Fgfr1^ind^*^−/−^ mice’s spinal cords compared to the control mice. *n* = 4/group. Data are expressed as the mean ± SEM. ns = not significant, * *p* < 0.05, ** *p* < 0.01.

To compare the effects of IFNβ-1a on FGF-dependent signaling in controls and *Fgfr1^ind^*^−/−^ mice, the proteins ERK/Akt/p38 and TrkB/BDNF were examined by western blot in the spinal cord. On day 62 post-EAE induction, *Fgfr1^ind^*^−/−^ mice on IFNβ-1a showed no regulation of FGFR1 protein expression (Figure 2A,B). There were no differences in the expression of the FGFR downstream molecules Akt and P38 or the TrkB receptor (Figure 2A,B). Compared with controls treated with IFNβ-1a, the increased phosphorylation of ERK (*p* < 0.01) and increased expression of BDNF (*p* < 0.05) were found (Figure 2A,B). The phosphorylation of STAT1 and STAT3 were not changed in *Fgfr1^ind^*^−/−^ (Figure 2A,B). These findings suggest that the effect on the signaling protein pERK and the neurotrophin BDNF are mediated by conditional FGFR1 gene deletion. 

### 2.3. Inhibition of FGFR1 and Application of IFNβ-1a Reduced Proliferation of Oli-Neu Oligodendrocytes

**Figure 3:**
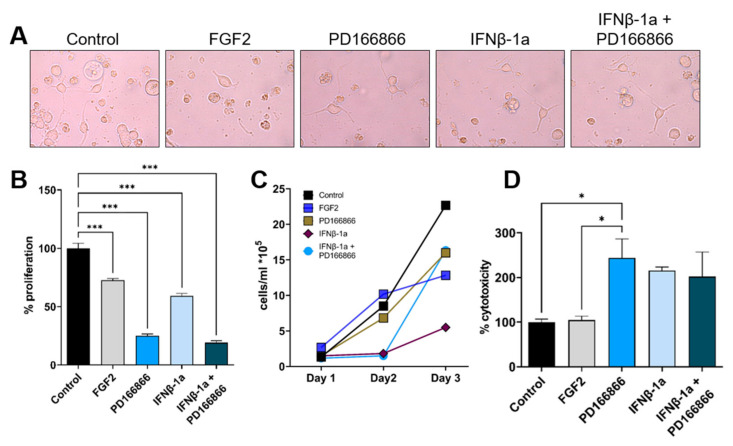
The proliferation and cytotoxicity effects of IFNβ-1a and FGFR1 inhibition by PD166866 in oligodendrocytes in vitro. Oli-neu oligodendrocyte cells were incubated for 24 h with culture medium containing FGF2 (20 ng/mL), PD166866 (10 µM), IFNβ-1a (400 ng/mL, Rabiff), IFNβ-1a + PD166866. (**A**–**C**) Proliferation were analyzed by counting the number of cells using light microscopy on day 1, 2, and 3. Cell counting assays showed FGFR1 inhibition by PD166866, IFNβ-1a, and IFNβ-1a + PD166866 decreases the proliferation of Oli-neu cells. (**D**) LDH cytotoxicity assays showed cytotoxicity effects of FGFR1 inhibition by PD166866. Representative microscopic images of Oli-neu cells under different treatments are shown in 20× magnification (**A**). Data are presented as mean ± SEM from three independent experiments, * *p* < 0.05, *** *p* < 0.001.

To study whether treatment of Oli-neu oligodendrocytes with the FGFR1 inhibitor PD166866 and/or IFNβ-1a alters the proliferation and cytotoxicity of oligodendrocytes, Oli-neu oligodendrocytes were treated with PD166866 (10 µM), IFNβ-1a (400 ng/mL, Rebif^®^) or combined IFNβ-1a and PD166866. Less proliferation of oligodendrocytes was found after FGFR1 inhibition (*p* < 0.001; Figure 3A,B), IFNβ-1a treatment (*p* < 0.001; Figure 3A,B) or a combined treatment of IFNβ-1a and FGFR1 inhibition (*p* < 0.001; Figure 3A,B) compared to controls. Increased cytotoxicity was observed after the application of PD166866 (*p* < 0.005; Figure 3D). 

### 2.4. IFNβ-1a Treatment along with FGFR1 Inhibition Results in Altered FGFR Downstream Signalling

**Figure 4:**
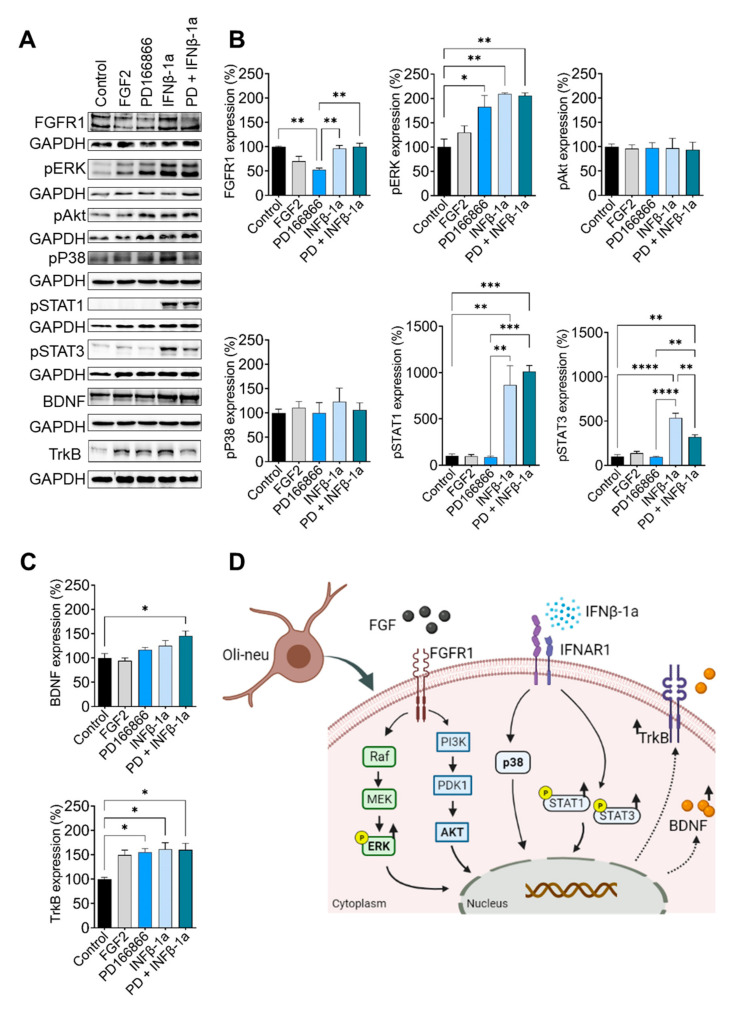
Effect of IFNβ-1a and FGFR1 inhibition by PD166866 in FGFR downstream signaling proteins in oligodendrocytes in vitro. Oli-neu oligodendrocyte cells were incubated with culture medium containing FGF2 (20 ng/mL), PD166866 (10 µM), IFNβ-1a (400 ng/mL, Rabiff), IFNβ-1a + PD166866. (**A**,**B**) FGFR1 protein expression was significantly lower in cells treated with PD166866 compared to controls. Protein expression of pERK, BDNF (**C**; PD with IFN) and TrkB were increased after PD166866, IFNβ-1a, and PD166866 with IFNβ-1a. Increased phosphorylation of STAT1 and 3 were found in cells treated with PD166866 as well as PD166866 with IFNβ-1a. (**D**) Overview of the effect of IFNβ-1a and FGFR1 inhibition by PD166866 in FGFR downstream signaling proteins in oligodendrocytes. Data are presented as mean ± SEM from three independent experiments, * *p* < 0.05, ** *p* < 0.01, *** *p* < 0.001, **** *p* < 0.0001.

To study whether FGFR1 inhibition and/or treatment with IFNβ-1a regulates FGFR downstream signaling in oligodendrocytes, we analyzed the phosphorylation of ERK/Akt/p38 and the expression of TrkB/BDNF. FGFR1 protein expression was lower after the application of PD166866 (*p* < 0.01) (Figure 4A,B). ERK phosphorylation was increased by FGFR inhibition (*p* < 0.05) and the combined treatment of PD166866 and IFNβ-1a (*p* < 0.01) (Figure 4A,B). Phosphorylation of Akt and p38 was not altered by any of the treatments (Figure 4A,B). STAT1 and STAT3 phosphorylation was increased by IFNβ-1a (pSTAT1: *p* < 0.05; pSTAT3: *p* < 0.001), and the combined treatment of IFNβ-1a and PD166866 (pSTAT1: *p* < 0.001; pSTAT3: *p* < 0.01 (Figure 4A,B). BDNF and TrkB expression was higher after the combined treatment of IFNβ-1a and PD166866 (BDNF: *p* < 0.05; TrkB: *p* < 0.05) (Figure 4A,C). Further, FGFR1 inhibition enhanced TrkB protein expression (*p* < 0.05). These in vitro results indicate that the combined treatment of IFNβ-1a and PD166866 alters FGFR1 signaling and increases the expression of pERK/TrkB/BDNF in Oli-neu oligodendrocytes.

## 3. Discussion

*Fgfr1^ind^*^−/−^ mice treated with IFNβ-1a showed less symptoms after the peak of disease and the chronic phase of EAE. Application of IFNβ-1a in controls had effects on symptoms at the peak of disease. The beneficial effects on the disease course in *Fgfr1^ind^*^−/−^ mice were associated with the activation of ERK phosphorylation and increased expression of the neurotrophin BDNF. In agreement with these EAE data, the in vitro findings showed that treatment of oligodendrocytes with IFNβ-1a or the FGFR1 inhibitor PD166866 enhances the phosphorylation of ERKand increases the expression of BDNF and its receptor TrkB. 

IFNβ-1a is a first-line treatment of relapsing MS (RMS) with anti-inflammatory effects in the periphery but not within the CNS [7]. In agreement with these facts, IFNβ-1a was effective at the peak of EAE, when immune cells cross over the blood–brain barrier and cause destruction of oligodendrocytes and myelin in the CNS [1,2]. IFNβ-1a and other substances given for multiple sclerosis have anti-inflammatory effects, reduce the number of relapses, and slow disease progression [3]. Neurodegeneration, which is mainly seen in the chronic phase of EAE, is more difficult to target. In progressive MS, neurodegeneration is the leading pathology, and to date, no substance significantly slows disease progression in these patients. 

In MS [11,25,26] and EAE models [9,15,16], FGF signaling pathways have been shown to be involved in the pathology. In MOG_35-55_-induced EAE, the conditional deletion of FGFR1 or FGFR2 in oligodendrocytes has a number of beneficial effects primarily in the chronic, neurodegenerative phase of EAE. In demyelinating spinal cord lesions, there are fewer lymphocyte and macrophage/microglia infiltrates, and myelin and axons are better preserved [9,15]. The beneficial effects seen in *Fgfr1^ind^*^−/−^ mice or *Fgfr2^ind^*^−/−^ mice were associated with changes in signaling molecules and the BDNF/TrkB pathway [9,14,15,27]. In the present study, the clinical findings in the chronic phase of EAE could be reproduced. 

To extend the knowledge on FGF signaling in EAE, controls and *Fgfr1^ind^*^−/−^ mice were both treated with a standard dose of IFNβ-1a from the time of EAE induction. The clinical findings as well as effects on the signaling cascade are similar to those of recent EAE experiments in *Fgfr1^ind^*^−/−^ mice or *Fgfr2^ind^*^−/−^ mice, suggesting that the findings are due to the conditional deletion of FGFR1 and are not a result of IFNβ-1a application. In this study, the reduction in disease severity in *Fgfr1^ind^*^−/−^ mice was accompanied by increased ERK phosphorylation and the increased expression of BDNF. In recent EAE experiments, downstream mediators of mitogen-activated protein kinases (MAPKs) such as ERK1/2 have been shown to regulate myelin formation. Mice without ERK1/2 activity show microglial activation, disturbed axonal integrity, and a partial loss of oligodendrocytes [28]. It is possible that the effects of phosphorylated ERK are mediated by BDNF signaling. Upregulated ERK and IP3/Akt signaling pathways enhance BDNF secretion, followed by the improved viability of neurons [29]. Investigations on the effects of recombinant BDNF on EAE support its neuroregenerative effects [30], whereas dysregulated BDNF secretion in MS patients is related with reduced neuroprotection [31]. Moreover, the increased expression of BDNF in T cells can be detected near demyelinating white matter lesions in EAE, implying that BDNF inhibits parenchymal injury and participates in neuroinflammatory responses during repair processes [32]. These data indicate that enhanced BDNF expression may be a reason for the ameliorated EAE disease course of *Fgfr1^ind^*^−/−^ mice.

Like in the EAE model, in vitro findings support the effects of FGFR modulation. The application of an FGFR inhibitor in oligodendrocytes resulted in enhanced BDNF/TrkB expression. It is important to note that BDNF/TrkB signaling is not only mediated by FGFR, but also belongs to the broad spectrum of IFNβ-1a-STAT signaling pathways. Under IFNβ-1a treatment, patients with RR-MS show elevated BDNF serum levels [33,34,35,36,37]. Further, in this study, yhe phosphorylation of STAT1/3 was increased by IFNβ-1a. IFNβ exerts its immunomodulatory, beneficial effects by the induction of T-reg cells and IL-10 secretion by binding to interferon receptors [38,39]. IFN-receptors, in turn, activate the Janus kinase (JAK) to phosphorylate STAT1 and STAT2 [39]. In contrast, in vivo, IFNβ-1a treatment does not regulate STAT1/3 and TrkB expression in the spinal cord at the chronic phase of EAE. The discrepancy/differences between the findings of protein expression from in vitro and in vivo experiments can be explained by (a) the duration of treatment: the time of IFNβ-1a administration in the EAE study and IFNβ-1a exposure in cell cultures, (b) time point: proteins were analyzed 62 days after IFNβ-1a administration and 24 h after IFNβ-1a exposure in cell cultures, (c) concentration: the presence of IFNβ-1a was different in the spinal cord compared to cells exposed in vitro. Besides the activation of the JAK/STAT pathway, recruitment of kinases such as PI3K, Akt, p38, JNK, and ERK corresponds to the increased ERK and BDNF expression in this study [40]. Taken together, EAE and in vitro findings result in increased pERK and BDNF signaling consequently creating a promyelinating and neuroprotective milieu in the CNS tissue, which manifests in an ameliorated EAE disease course.

In summary, this study does not provide evidence for a crosstalk mechanism between IFNβ-1a and FGFR1 signaling. FGFRs may be a potential target for therapy of EAE and patients with MS and may be more promising than treatment with interferon beta-1a.

## 4. Materials and Methods

### 4.1. Ethics Statement and Housing Conditions

All scientific procedures on animals were approved by the regional council of Hesse, Giessen, Germany (20/23-Nr. 31/2008) in accordance with the German Animal Welfare Act and the European legislation for the protection of animals used for scientific purposes (2010/63/EU). AVMA guidelines for euthanasia of animals were followed. All efforts were made to minimize pain and suffering. All mice were housed in a controlled environment and kept to a 12 h light/dark cycle. Mice had free access to a standard pellet diet (Altromin 1324 TPF, Altromin Spezialfutter GmbH, Lage, Germany) and autoclaved water ad libitum. 

### 4.2. Generation of FGFR1 Conditional Knockout Mice

*Fgfr1^ind^*^−/−^geflox flox (B6.129S4-*Fgfr1^tm5^.^1Sor^*/J), Plp-creERT (B6.Cg-Tg(Plp1-cre/ER^T^)3Pop/J) and C57BL/6 mice (Mus musculus) were purchased from The Jackson Laboratories (Bar Harbor, ME, USA). *Fgfr1^ind^*^−/−^geflox flox (B6.129S4-*Fgfr1^tm5^.^1Sor^*/J) mice were crossbred with Plp-creER^T^ (B6.Cg-Tg(Plp1-cre/ER^T^)3Pop/J) mice and backcrossed to C57BL/6J (Final mice line: B6.Cg-Tg(Plp1-cre/ER^T^)3Pop *Fgfr1^tm5^.^1Sor^*). The generation of conditional FGFR1 knockout mice was achieved as described earlier [15,16]. Briefly, the genomic DNA was isolated (DirectPCR-Tail, Peqlab, Erlangen, Germany) to identify the deletion of the FGFR1 floxed allele and PLP transgene, and mice were genotyped by PCR. For the induction of Cre recombinase, 4-week-old B6.Cg-Tg(PLP1-cre/ER^T^)3Pop *Fgfr1^tm5^.^1Sor^* female mice were administered with 1 mg of tamoxifen (Sigma-Aldrich, Steinheim, Germany) in 100 µL sunflower oil/ethanol i.p. over 5 consecutive days (referred to as *Fgfr1^ind^*^−/−^). B6.Cg-Tg(PLP1-cre/ERT)3Pop *Fgfr1^tm5^.^1Sor^* littermate control mice received a sunflower oil/ethanol mixture (referred to as controls).

### 4.3. EAE Induction and Evaluation of Symptoms 

EAE induction and evaluation of symptoms were performed as described earlier [15]. Briefly, EAE was induced in 8-to-12-week-old female *Fgfr1^ind^*^−/−^ and control mice by subcutaneous injection of 300 µg myelin oligodendrocyte glycoprotein peptides (MOG_35-55_; Charité Hospital, Berlin, Germany) emulsified in complete Freund’s adjuvant (Sigma, Steinheim, Germany) containing 10 mg Mycobacterium tuberculosis (Difco, Detroit, MI, USA). Intraperitoneal injections of 300 ng pertussis toxin (Calbiochem, Darmstadt, Germany) were given at the time of immunization and 48 h later. The EAE disease course was monitored until day 62 in a blinded manner. Mice were euthanized at day 62 p.i. for the chronic phase. Mice were evaluated daily in a blinded fashion using the following 5-scale score criteria: 0 to 5 where 0 = normal, 0.5 = distal tail weakness, 1 = complete tail weakness, 1.5 = mild hind limb weakness, 2 = ascending hind limb weakness, 2.5 = severe hind limb weakness, 3 = hind limb paralysis, 3.5 = hind limb paralysis and moderate forelimb weakness, 4 = hind limb paralysis and severe forelimb weakness, 4.5 = tetraplegia and incontinence, and 5 = moribund/death. 

### 4.4. Administration of IFNβ-1a

IFNβ-1a (Rebif^®^) was kindly provided by Merck Serono GmbH, Darmstadt, Germany. In this study, we used three groups of mice: control, IFNβ-1a alone (referred as IFNβ-1a), and IFNβ-1a treated *Fgfr1^ind^*^−/−^ mice (referred as *Fgfr1^ind^*^−/−^ IFNβ-1a). IFNβ-1a (30 mg/kg) were subcutaneously injected daily from day 0 to day 7 after induction of EAE (Figure 1A). 

### 4.5. In Vitro Oli-Neu Oligodendrocyte Cultures

Oli-neu oligodendrocyte adherent cell lines were used in this study. Cells were cultured at 37 °C in 5% CO_2_. The Oli-neu cell line was maintained in DMEM medium supplemented with B27 supplement (Invitrogen), 0.011% sodium pyruvate, 500 nM Tri-lodo-L-Thyronine, 520 nM L-Thyroxine. Poly-L-Lysine was coated in a 75 cm^2^ culture flask for at least 5 h or overnight, and washed with 1X PBS for 3 times before use. Oli-neu cells were seeded in the Poly-L-Lysine coated flask, and above 80% confluent flasks were used for experiments. 

### 4.6. FGFR1 Inhibitor PD166866 and Application of Interferon Beta 1a in Oli-Neu Cells In Vitro

PD166866, a potent FGFR1 tyrosine kinase inhibitor, was used in this study. PD166866 was purchased from Sigma-Aldrich (Steinheim, Germany) and dissolved in DMSO, and above 80% confluent cells were treated with a 10 µM concentration of PD166866 for 3 h. Effects of IFNβ-1a and FGFR1 inhibition on proliferation, cytotoxicity, FGFR signaling, and TrkB/BDNF protein expression in Oli-neu oligodendrocytes were analyzed. Oli-neu culture was performed as described previously [41]. Briefly, experiments were performed at passages 8–12. To assess the effects of IFNβ-1a and FGFR1 inhibition on cell proliferation, FGFR signaling, and myelin proteins, Oli-neu cells were treated with FGF2 (20 ng/mL), PD166866 (10 μM), IFNβ-1a (400 ng/mL, Rebif^®^), IFNβ-1a + PD166866, and DMSO. For FGFR signaling activation, stimulation with FGF2 was performed. After 24 h of incubation, protein isolation and western blot were carried out [41]. 

### 4.7. Protein Extraction and Western Blot Analysis

Spinal cord tissue was homogenized in lysis buffer with Tissue ruptor (Qiagen Instruments, Hombrechtikon, Switzerland). In vitro experiments were performed upon respective treatments, cells were scraped from the flask, cell pellets were lysed, cell lysates were centrifuged at 14,000 rpm in 4 °C for 30 min, and proteins were collected and stored at −20 °C. Protein concentrations were quantified (Pierce^®^ BCA Protein Assay Kit, Thermo Scientific, Rockford, IL, USA) and normalized. Thirty micrograms of proteins were fractionated by denaturing gel electrophoresis (10% SDS-PAGE), transferred (Trans Blot, Semi dry Transfer cell, BioRad, Hercules, CA, USA) to a nitrocellulose membrane (GE Healthcare, AmershamTM Hybond ECL, Buckinghamshire, UK), and blocked with 5% BSA for 1 h. The membranes were incubated overnight at 4 °C with target-protein-specific primary antibodies, followed by 1 h incubation with respective secondary antibodies (Table 1). The immunoreactive bands of target proteins were detected with SuperSignal West Pico Chemiluminescent Substrate (Thermo Scientific, Rockford, IL, USA) using an ECL ChemoCam Imager (INTAS Science Imaging Instruments GmbH, Göttingen, Germany). GAPDH was used as a loading control. Protein band densities were analyzed by ImageJ 1.53b software (National Institute of Health, Bethesda, AR, USA). 

### 4.8. Proliferation Assays 

To assess the effects of IFNβ-1a and FGFR1 inhibition on the proliferation of Oli-neu oligodendrocytes, a WST proliferation assay and tryphan blue staining were utilized. Oli-neu oligodendrocytes were seeded and maintained in culture until the cells tightly adhered to the bottom of the plates. Cells were incubated for 24 h with fresh medium containing FGF2 (20 ng/mL), PD166866 (10 µM), IFNβ-1a (400 ng/mL, Rebif^®^), and IFNβ-1a + PD166866. After 24, 48, and 72 h of incubation, manual counting was performed using a Neubauer improved chamber (Karl Hecht “Assistant”, Altnau TG, Switzerland) and trypan blue dye (Carl Roth, Karlsruhe, Germany) as described by others [42]. For photometric evaluation of proliferation, cells were seeded in a final volume of 100 µL/well (1 × 10^5^ cells/mL) before treatment. After 24 h of treatment, a WST-1 assay (Roche Applied Science, Mannheim, Germany) was carried with 96-well plates (Greiner Bio-One, Frickenhausen, Germany). Absorption was measured by an ELISA-Reader (Multiscan EX, Thermo Fisher Scientific, Langenselbold, Germany) at a wavelength of 405 nm using a reference wavelength of 492 nm.

### 4.9. Cytotoxicity Assay 

Cytotoxic effects of IFNβ-1a and FGFR1 inhibition in Oli-neu oligodendrocytes were studied by the measuring of lactate dehydrogenase (LDH) in the supernatant of incubated cells (as described in proliferation assay) (96-well plate, 0.5 × 10^4^ cells/well) using a prefabricated kit (Cytotoxicity Detection Kit (LDH), Roche Diagnostics, Mannheim, Germany). Cell plates were centrifuged for 10 min at 250× *g*, and the supernatant was used to measure absorbance at 492 nm (reference wavelength: 620 nm) to calculate cytotoxicity.

### 4.10. Quantification and Statistical Analysis

All analyses were performed in a blinded fashion. EAE scores were analyzed using a two-way ANOVA with Tukey’s post hoc test. Western blot data were analyzed using a *t*-test. Statistical analysis and graph preparation was performed using GraphPad Prism 9 (GraphPad Software, San Diego, CA, USA). Graphical displays were prepared using BioRender web application. Data are expressed as the mean ± standard error of mean (SEM). * indicates *p* ≤ 0.05, ** indicates *p* ≤ 0.01, *** indicates *p* ≤ 0.001.

## Figures and Tables

**Table 1:** List of antibodies used for Western blot.

Name	Host	Mol. Weight	Method	Art. No	Manufacturer
**Primary Antibodies**					
Anti-pERK	Rabbit	44, 42 kDa	WB	4370s	Cell Signaling Tech., Danvers, MA, USA
Anti-pAkt	Rabbit	60 kDa	WB	4060s	Cell Signaling Tech., Danvers, MA, USA
Anti-pP38	Rabbit	43 kDa	WB	9212s	Cell Signaling Tech., Danvers, MA, USA
Anti-pSTAT1	Rabbit	84, 91 kDa	WB	9171s	Cell Signaling Tech., Danvers, MA, USA
Anti-pSTAT3	Rabbit	79, 86	WB	9145s	Cell Signaling Tech., Danvers, MA, USA
Anti-FGFR1	Rabbit	110 kDa	WB	sc-57132	Santa Cruz Biotech., Dallas, CA, USA
Anti-Trk B	Rabbit	145 kDa	WB	sc-377218	Santa Cruz Biotech., Dallas, CA, USA
Anti-BDNF	Rabbit	14 kDa	WB	sc-65514	Santa Cruz Biotech., Dallas, CA, USA
Anti-GAPDH	Mouse	37 kDa	WB	sc-365062	Santa Cruz Biotech., Dallas, CA, USA
**Secondary Antibodies**					
Anti-rabbit-HRP	Goat			7074	Cell Signaling Tech., Danvers, MA, USA
Anti-mouse-HRP	Horse			7076	Cell Signaling Tech., Danvers, MA, USA

## Data Availability

The data presented in this study are available upon request to corresponding author.
